# Cardiovascular Complications of Viral Respiratory Infections and COVID-19

**DOI:** 10.3390/biomedicines11010071

**Published:** 2022-12-27

**Authors:** Paweł Franczuk, Michał Tkaczyszyn, Maria Kulak, Esabel Domenico, Piotr Ponikowski, Ewa Anita Jankowska

**Affiliations:** 1 Institute of Heart Diseases, Wroclaw Medical University, 50-556 Wroclaw, Poland; 2 Institute of Heart Diseases, University Hospital, 50-556 Wroclaw, Poland; 3 Faculty of Medicine, Medical University of Gdansk, 80-210 Gdansk, Poland; 4 Faculty of Medicine, Wroclaw Medical University, 50-345 Wroclaw, Poland

**Keywords:** respiratory viruses, respiratory infection, cardiovascular disease, myocarditis, COVID-19, heart failure

## Abstract

Viral respiratory infections (VRI) are the most prevalent type of infectious diseases and constitute one of the most common causes of contact with medical care. Regarding the pathophysiology of the cardiovascular system, VRI can not only exacerbate already existing chronic cardiovascular disease (such as coronary artery disease or heart failure) but also trigger new adverse events or complications (e.g., venous thromboembolism), the latter particularly in subjects with multimorbidity or disease-related immobilization. In the current paper, we provide a narrative review of diverse cardiovascular complications of VRI as well as summarize available data on the pathology of the circulatory system in the course of coronavirus disease 2019 (COVID-19).

## 1. Introduction

Viral respiratory infections (VRI) are among the most common reasons of contact with health care in both adults and children [1]. From a global perspective, respiratory infections are the most prevalent type of infectious diseases and one of the leading causes of death, following only ischemic heart disease, chronic obstructive pulmonary disease, and stroke, and are responsible for 120 million disability-adjusted life years worldwide [2,3,4]. Viral etiology remains the most common in both respiratory infections in total and also in the subgroup of subjects with pneumonia—the most severe type of infectious involvement of the respiratory tract [5,6].

**Figure 1:**
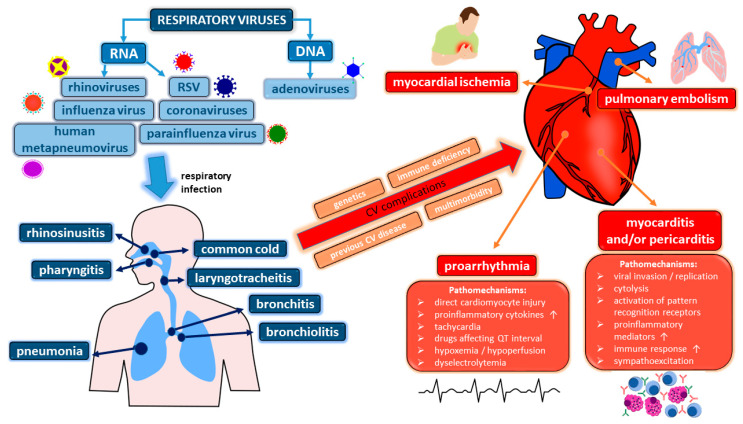
In predisposed patients, common upper and lower respiratory tract infections of viral etiology may be complicated by adverse cardiovascular events and conditions, the pathophysiology of which is diverse and not always thoroughly investigated. The pathomechanisms of myocardial ischemia and pulmonary embolism are presented in Figure 2. Abbreviations: CV—cardiovascular, DNA—deoxyribonucleic acid, RNA—ribonucleic acid, RSV—respiratory syncytial virus.

The most frequently identified viruses in patients with acute presentations of VRI are influenza virus, rhinoviruses, respiratory syncytial virus (RSV), parainfluenza virus, human metapneumovirus, respiratory adenoviruses, and coronaviruses [1,7,8,9]. However, it needs to be acknowledged that multiple viral pathogens are found in many subjects [10]. Respiratory viruses are transmitted predominantly via inhalation of infectious droplets or contact with contaminated secretions [1]. Clinical manifestations of VRI are heterogeneous and may involve the upper and/or lower respiratory tract, comprising rhinosinusitis, pharyngitis, the common cold, laryngotracheitis, bronchitis, bronchiolitis, and eventually overt pneumonia [11]. From the point of view of the cardiovascular system, VRI may not only exacerbate already existing chronic cardiovascular disease (such as coronary artery disease or heart failure) but also trigger new adverse cardiovascular events/conditions, the latter particularly in subjects with multimorbidity or immune deficits (Figure 1). Currently, due to the latest research boosted by the coronavirus disease 2019 (COVID-19) pandemic, our knowledge of and interest in the pathophysiology of VRI have considerably increased. In the current paper we provide a narrative review on diverse cardiovascular complications of VRI, as well as a summary of available data on the involvement of the cardiovascular system in the course of COVID-19.

## 2. Ischemic Complications

From a pathophysiological point of view, myocardial ‘ischemia’ results from an imbalance between myocardial oxygen demand/supply, whereas myocardial ‘injury’ is defined as any damage to myocardial cells that is accompanied by the release of cardiac necrotic biomarkers [12]. It needs to be acknowledged that the prevalence of myocardial infarction (MI) varies seasonally and is highest in the winter [13]. The number of daily hospitalizations rises starting in August and reaches its peak in January [13]. This seasonality is not fully explained, but the increased incidence of upper respiratory tract infections is considered to play a role due to their multifaceted impact on blood rheology and therefore the functioning of the cardiovascular system [13]. The total risk for cardiovascular complications is determined primarily by the severity of the respiratory infection [14,15]. Although cardiac troponins above the upper limit of normal are frequently detected in the peripheral blood of patients with ongoing severe VRI [16,17], the most frequent pathomechanism is presumably the direct/indirect influence of the viruses themselves on cardiomyocytes (myocardial injury) [14].

The risk of MI during the first week following VRI is significantly increased (up to 6-fold) and remains elevated during one month of observation in Scottish records of the 10-year national infections registry, including 1989 individuals with acute ischemic cardiovascular events [18]. Similar trends have also been demonstrated for incident stroke, and therefore it should be assumed that the increased risk should be attributed to the entire spectrum of atherosclerotic cardiovascular disease (ASCVD). It is worth noting that the risk of an acute cardiovascular event was also related to previous ASCVD burden (the highest in patients with a history of previous MI) and the type of infection (the highest in influenza) [19,20]. There is evidence from longitudinal observations for an increased risk for ASCVD-related morbidity and mortality (including MI, stroke, or cardiovascular death) for a 10-year period following the hospitalization for any severe or non-severe pneumonia [21].

**Figure 2:**
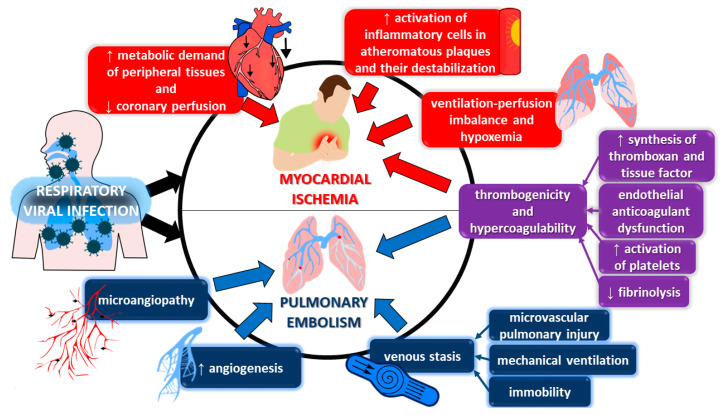
Multifaceted and overlapping mechanisms of arterial ischemic and venous thromboembolic complications of viral respiratory infections.

There are several pathophysiological links between VRI and the triggering or worsening of myocardial ischemia, including (i) inflammation; (ii) prothrombotic imbalance; (iii) hypercoagulability; and (iv) increased metabolic demands of the myocardium [22,23,24] (Figure 2). The process of inflammation not only activates platelets but also stimulates inflammatory cells within atherosclerotic plaques. The latter results in the release of metalloproteinases and peptidases, which can contribute to plaque destabilization [23,24]. Moreover, circulating pro-inflammatory cytokines can negatively impact the process of atherosclerotic remodeling of the vessel wall through modulating monocyte adhesion, macrophage activation, and proliferation of smooth muscle cells [24]. In parallel, up-regulated synthesis of thromboxane and tissue factor expression on immune cells, as well as the impairment of fibrinolysis and anticoagulant function of the endothelium, lead to increased thrombogenicity and a hypercoagulability state [24]. The anticoagulant dysfunction is associated with the downregulation of protein C, while the disturbance of fibrinolysis is associated with an increase in plasminogen activator inhibitor 1 [24].

## 3. Thromboembolism

Infections per se augment the risk of venous thromboembolism (VTE) up to about two times in records from over 10,000 individuals with urinary or respiratory tract infections [25]. Respiratory infections are associated with increased risk of both components of VTE, analyzed separately: deep vein thrombosis (DVT) and pulmonary embolism (PE) [25]. The overlapping of symptoms between PE and respiratory infection makes it frequently difficult to establish the chronology of these clinical entities in clinical practice [25].

The established pathomechanisms linking infection and thromboembolism include not only platelet activation and up-regulated synthesis of pro-coagulant proteins but also the impairment of fibrinolysis and anticoagulant function of the endothelium [26] (Figure 2). Moreover, the activation of leukocytes, triggered by infection, is associated with the release of damage-associated molecular patterns (such as deoxyribonucleic acid or histones), which further promotes thrombus formation [26]. It also needs to be acknowledged that local inflammatory reactions, for example in the lungs, result in the disruption of endothelial cell membranes, which is followed by vascular thrombosis, microangiopathy, and increased angiogenesis [27,28]. Venous stasis, which is prevalent in immobile critically ill patients undergoing mechanical ventilation and presenting with microvascular pulmonary injury, is another pathomechanism increasing thromboembolic events in such patients [29].

## 4. Viral Myocarditis and (Post-)Inflammatory Cardiomyopathy

Despite many new experimental studies, our understanding of the development, evolution/progression, and recovery (or not) from an acute/sub-acute myocarditis is still not sufficient, and several pathways/mechanisms are constantly being explored, including the role of immune cells (also autoimmunity), the pathobiology of particular viruses, and iron metabolism, to name but a few [30,31,32,33]. Respiratory viruses are established to be the most common triggers of myocarditis, and the most frequently identified/isolated ones are adenoviruses, enteroviruses, influenza virus, and coronaviruses [30,34,35]. The incidence of particular viral infections fluctuates seasonally, with the peak of influenza in the winter and enteroviruses in the summer and autumn [30]. Clinical presentation of viral myocarditis is heterogeneous and comprises a broad spectrum of symptoms, from chest pain (ischemic-like or pleuritic-like), dyspnea, and fatigue, through less specific palpitations or syncope, to fulminant life-threatening conditions such as cardiogenic shock, ventricular arrhythmias, or even sudden cardiac death [36].

Adenoviruses and enteroviruses are positioned among the most common etiological factors of myocarditis [34]. They represent a group of primary cardiotropic viruses, responsible for direct damage to myocardial tissue. Viral invasion into cardiomyocytes occurs via the transmembrane receptor and is followed by viral replication inside, leading to the destruction of the cytoskeleton, cytolysis, and eventually an immune cell reaction [30,37,38]. Persistent viral activity following the acute phase of the disease can result in progressive cardiac dysfunction with a poor prognosis [35]. There is evidence that, for example, in enteroviral myocarditis, the recovery from an acute condition defined as complete virus clearance occurs in only half of subjects [39]. In this context, it is worth noting that entero- or adenoviral genomes were detected in 26% of patients with idiopathic left ventricular dysfunction and in 13% of patients with idiopathic dilated cardiomyopathy. However, it should be stipulated that this does not prove causality [35,40].

Influenza A and influenza B viruses (together with the Coronaviridae family described below) are classified as cardiotoxic agents, provoking myocarditis indirectly; these viruses activate the immune system responses, leading to augmented cytokine release and cytokine-mediated myocardial damage [30,41]. Influenza myocarditis is considered infrequent but associated with a poor outcome, with a mortality rate ranging up to 30% in H1N1 subtype infections [41].

## 5. Pericardial Disease

In developed countries, viral etiology is the most common in both acute pericarditis and pericardial disease as a whole [42,43]. Acute pericarditis is an inflammatory disease characterized by infiltrates of immune cells into the pericardium triggered mainly by viruses and resulting in a clinical syndrome characterized by typical signs and symptoms (pericarditic type of chest pain, specific electrocardiogram (ECG) abnormalities, and pericardial effusion) [42,44]. The common course of the disease is benign with mild to moderate symptoms that can be successfully treated outpatiently with non-steroidal anti-inflammatory agents and colchicine. More severe complications, including cardiac tamponade with a worse prognosis, are rather rare [44,45]. 33% of patients with acute pericarditis have a history of a recent upper respiratory tract infection [46]. Among respiratory viruses, enteroviruses, adenoviruses and influenza virus are the most prevalent in patients with pericarditis, being identified in 25%, 19%, and 6% of patients, respectively [47]. Differences in the clinical course of the disease according to particular groups of viruses have not been investigated. Not infrequently, pericarditis is accompanied by the involvement of myocardial muscle, which results in a syndrome of ‘myopericarditis’ [47,48]. This condition is associated with enteroviral infection in 15% and adenoviral, influenza, or parainfluenza in 10% each [47,48].

## 6. Pro-Arrhythmia

Diverse cardiovascular and non-cardiovascular factors (the latter including, for example, dehydration and electrolyte disturbances caused by hyperthermia or diarrhea) contribute to pro-arrhythmia in the course of VRI. Not surprisingly, the patients most vulnerable to severe arrhythmic complications are those who already have a chronic cardiovascular disease (such as heart failure or non-revascularized coronary artery disease) that may be a substrate for life-threatening ventricular arrhythmias [30,49,50]. Obviously, both supraventricular and ventricular arrhythmias can also be triggered by direct (e.g., cardiomyocyte invasion in acute myocarditis complicating VRI) or indirect (e.g., in the course of excessive pro-inflammatory cytokine release) myocardial injury associated with an infection that is initially limited to the respiratory system [30]. Regarding the impact of severe respiratory infection on the functioning of the cardiovascular system, it also needs to be acknowledged that coexistent (sub-)acute myocardial ischemia (resulting from, e.g., hypoxemia, hypoperfusion, or tachycardia, to name but a few) can also promote arrhythmic episodes [22,23,24].

Epidemiological data show interesting trends: seasons with higher influenza activity are characterized by an increased risk of device-detected ventricular arrhythmias treated with appropriate therapies in patients with implantable cardioverter-defibrillators [51]. In one analysis of a large cohort of patients, it has been demonstrated that the prevalence of ventricular arrhythmia requiring high-energy discharge or antitachycardia pacing therapy followed the community activity of the influenza virus [51]. This relationship can be potentially explained by inflammation, exacerbation of heart failure or coronary artery disease, and increased myocardial oxygen demand [51]. Furthermore, among adult patients hospitalized due to RSV infection, 8% developed a new arrhythmia, and after considering all cardiovascular complications, only exacerbation of heart failure symptoms was slightly more frequent in this group of subjects [52].

Life-threatening ventricular arrhythmias or severe cardiac conductance disorders are most likely to occur in the course of giant-cell myocarditis [30,49,53]. The pathomechanism of significant (ventricular) arrhythmias in patients with (sub-)acute myocarditis is complex and there is evidence on the contribution of direct viral invasion within cardiomyocytes, microvascular ischemia, proarrhythmic properties of particular cytokines, abnormal calcium handling, and deranged ion channel functioning [54,55]. Pericarditis can also be complicated by arrhythmias, although it is considerably less prevalent than myocarditis [48].

## 7. Coronaviruses and Severe Acute Respiratory Syndrome Coronavirus 2

Although common coronaviruses are an etiological factor for mild respiratory infections [56], a few particular coronaviruses responsible for epidemic outbreaks occurring in the last two decades (Severe Acute Respiratory Syndrome Coronavirus 1 (SARS-CoV-1—2002, China); Middle East Respiratory Syndrome Coronavirus (MERS-CoV—2012, Saudi Arabia)) have been linked to greater morbidity and mortality in humans [15]. Although 20 years have elapsed since the outbreak of severe acute respiratory syndrome (SARS), our knowledge regarding this pathogen is still based on small cohorts’ descriptions [15,57]. The most prevalent cardiovascular symptoms in patients with SARS-CoV-1 were tachycardia (72%) and hypotension (50%) [57,58]. The echocardiographic evaluation of 46 patients revealed transient diastolic dysfunction without systolic impairment in the entire group of infected subjects, whereas in patients who required mechanical ventilation, a decrease in left ventricular ejection fraction was noted [59]. Cases of MI and PE have also been documented during SARS-CoV-1 infection [60]. The Middle East respiratory syndrome (MERS) epidemic occurred 10 years after SARS, and there are only anecdotal records on how it affects the cardiovascular system or impacts concomitant chronic cardiovascular disease [61].

**Figure 3:**
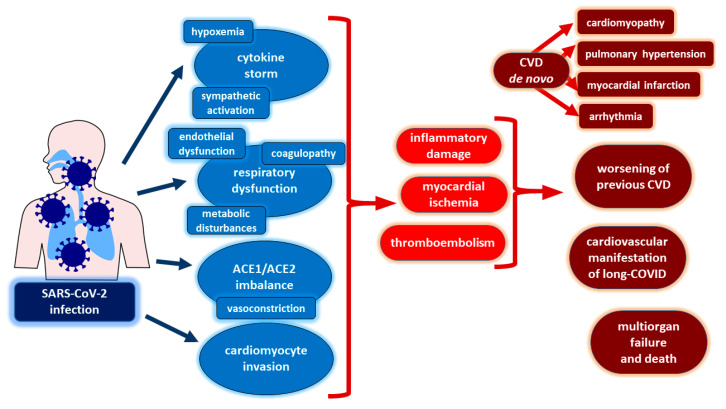
Cardiovascular complications of COVID-19 and diverse proven underlying pathomechanisms. Abbreviations: ACE1—angiotensin-converting enzyme 1, ACE2—angiotensin-converting enzyme 2, CVD—cardiovascular disease, SARS-CoV-2—Severe Acute Respiratory Syndrome Coronavirus 2.

We have much more high-quality data for Severe Acute Respiratory Syndrome Coronavirus 2 (SARS-CoV-2) due to the global impact of the COVID-19 pandemic and the great social need for large-scale research. The multidimensional impact of SARS-CoV-2 infection on the human organism has been demonstrated in both experimental studies and patient data analyses. Due to the pleiotropic effects of SARS-CoV-2 infection, the spectrum of COVID-19 complications is wide and heterogeneous and without doubt requires a multidisciplinary approach. For example, COVID-19 has been linked with neurological and psychiatric conditions (stroke, encephalitis, psychosis), reproductive system disturbances in males (infertility, erectile dysfunction), and gestational complications in females (preeclampsia, hypertension) [62,63,64]. With regard to the circulatory system, analogously to other respiratory viruses, SARS-CoV-2 not only can exacerbate (decompensate) pre-existing cardiovascular disease (such as heart failure), but in predisposed patients it may also be responsible for new adverse cardiac events (Figure 3). Acute myocardial injury as reflected by elevated cardiac troponin levels is found in 7–36% of COVID-19 patients and correlates with increased in-hospital mortality and the need for mechanical respiratory support [65,66,67,68,69,70,71]. Moreover, individual cases of fulminant heart failure with severe systolic dysfunction have also been published [72,73].

There are a few different pathomechanisms of myocardial injury in patients with COVID-19. They include, for example, direct myocardial damage due to uncontrolled cytokine release [67,74] or myocardial ischemia triggered by respiratory dysfunction with hypoxemia in the course of severe respiratory insufficiency [75]. Furthermore, ACE2 downregulation by interfering with the balance between angiotensin-converting enzyme 1 (ACE1) and angiotensin-converting enzyme 2 (ACE2) can lead to the suppression of the cardioprotective, anti-inflammatory, and vasodilative effects of the ACE2–angiotensin axis [76]. Additional pathomechanisms contributing to myocardial injury in COVID-19 are endothelial dysfunction, coagulopathy, and metabolic disturbances with insulin resistance and pericardial lesions [75,77]. SARS-CoV-2 is proven to directly invade cardiomyocytes in vitro in a cathepsin- and ACE2-dependent way [78]. Regarding autopsy studies, high cardiac viral loads were found in 41% of subjects [79] and interstitial infiltrates of mononuclear immune cells, macrophages with viral particles, and endotheliitis were demonstrated [72,80,81].

There have been documented cases of acute myocarditis in the course of symptomatic COVID-19 [73,82]. However, the causal relationship has not been fully elucidated, and the prevalence of this coincidence is not precisely established [65]. Among patients recovered from recent COVID-19, in magnetic resonance imaging (MRI), cardiac involvement was found in 78% of subjects, while myocardial inflammation was present in 60% of enrollees. It is worth noting that cardiac magnetic resonance (CMR) abnormalities are more prevalent than abnormal cardiac biomarkers in patients with a recent history of COVID-19 [83,84,85]. In the recent meta-analysis, heart failure symptoms were described in 11.5% of patients with COVID-19 and were related to higher mortality [86]. Analogously, elevated natriuretic peptides predict increased mortality in patients hospitalized with COVID-19 [87].

COVID-19 is related to an increased risk for ischemic complications. The risk of MI is five times higher and the risk of stroke is ten times higher during the first 2 weeks after diagnosis, and persists for at least 1 month [88]. MI is diagnosed in 7–17% of COVID-19 inpatients and may have a heterogeneous etiology: plaque rupture, spasm of the coronary artery, micro-thrombi or insufficient oxygen supply due to hypoxemia (type 2 MI according to guidelines) or endothelial or vascular injury [89]. The incidence of ischemic stroke in hospitalized patients reaches 2.5–5% [90], and cases of aortic thrombosis, acute limb, or mesenteric ischemia have also been described [91]. Thromboembolic events are prevalent and related mainly to hyperinflammatory reactions and microvascular dysfunction [67,92,93,94]. VTE occurs in 29-37% of subjects (predominantly PE—18%), with a demonstrated reduction to 24% (15% in the case of PE) when antithrombotic prophylaxis is implemented [27]. In autopsy, alveolar capillary microthrombi in COVID-19 patients are almost 10 times more frequent than in influenza [28]. VTE is considerably more prevalent in ARDS associated with COVID-19 than in non-COVID-19 ARDS [95]. Furthermore, arrhythmias affect almost every fifth patient and are also related to worse outcomes [86]. However, arrhythmic burden is not associated with the severity of lung injury [96,97]. New-onset atrial fibrillation was found in up to 6% of patients hospitalized due to COVID-19 [66,97,98].

Long-COVID (persistence of signs or symptoms over 4 weeks from acute onset) consists of two stages: ongoing symptomatic phase (4–12 weeks) and post-COVID-19 syndrome (>12 weeks), and is manifested in 43–89% of the patients by cardiopulmonary symptoms (chest pain, breathlessness, palpitations, and fainting) [99,100,101]. In one study, at least 4 months after COVID-19 the convalescents had higher troponin and N-terminal pro-brain natriuretic peptide (NT-proBNP) levels together with slight biventricular contractile dysfunction as assessed by echocardiography in comparison to non-COVID controls [102].

Although SARS-CoV-2 infection in children is usually mild or asymptomatic, a rare but severe complication among the youngest constitutes multisystem inflammatory syndrome in children (MIS-C; also described as pediatric inflammatory multisystem syndrome, or PIMS), which in the majority of cases affects the heart and coronary arteries [103,104]. The postulated underlying mechanisms are immune-driven, induced by a hyperimmune response to the virus in genetically vulnerable subjects [103]. The most prevalent symptoms are persistent fever and gastrointestinal manifestations, while the most common cardiac complications include left ventricular systolic dysfunction, coronary artery aneurysms, and electrical abnormalities (arrhythmias or conduction disturbances) [103]. The described complications are similar to Kawasaki disease, toxic shock syndrome, macrophage activation syndrome, bacterial sepsis, and cytokine release syndrome, which only confirms their common immunological denominator [103,105]. In severe cases requiring inotropic agents, mechanical ventilation or extracorporeal membrane oxygenation (ECMO) are required. Troponin elevation can be found in 64-95% of children with MIS-C [103,106]. The majority of patients recover within several weeks, with the mortality rate estimated at approximately 2%. However, the long-term consequences have not been elucidated yet [103,104,105,106]. Established risk factors for poor prognosis comprise older age and high serum ferritin [107].

## 8. Conclusions

Some cardiovascular complications of VRI are well characterized; for example, we know in detail how such infections adversely affect blood rheology or endothelial function. In the course of, for example, COVID-19, pathophysiological aspects such as hypoxemia due to severe respiratory failure or a cytokine storm have also been intensively studied in the context of the impact on the circulatory system. On the other hand, we still do not know much about what happens at the virus-cardiomyocyte level, i.e., which factors determine, for example, the development of acute/sub-acute myocarditis (complicating a common viral upper respiratory tract infection) or the conversion of acute inflammatory myocardial involvement to chronic post-inflammatory cardiomyopathy. A more precise understanding of these pathomechanisms will not only allow us to identify more precisely subjects at risk of the most severe VRI cardiovascular complications (cardiomyopathy with severe symptoms of heart failure), but it may also allow us to develop effective causal or even prophylactic anti-inflammatory/immunosuppressive therapies, which in a carefully selected group of patients may reduce morbidity and mortality.
